# Construction of MoS_2_-ReS_2_ Hybrid on Ti_3_C_2_T_x_ MXene for Enhanced Microwave Absorption

**DOI:** 10.3390/mi14111996

**Published:** 2023-10-27

**Authors:** Xiaoxuan Xu, Youqiang Xing, Lei Liu

**Affiliations:** 1 School of Business and Trade, Nanjing Vocational University of Industry Technology, Nanjing 210023, China; xuxx@niit.edu.cn; 2 Jiangsu Key Laboratory for Design and Manufacture of Micro-Nano Biomedical Instruments, School of Mechanical Engineering, Southeast University, Nanjing 211189, China

**Keywords:** interface engineering, microwave absorption, Ti_3_C_2_Tx MXene, transition metal disulfides

## Abstract

Utilizing interface engineering to construct abundant heterogeneous interfaces is an important means to improve the absorbing performance of microwave absorbers. Here, we have prepared the MXene/MoS_2_-ReS_2_ (MMR) composite with rich heterogeneous interfaces composed of two-dimensional Ti_3_C_2_Tx MXene and two-dimensional transition metal disulfides through a facile hydrothermal process. The surface of MXene is completely covered by nanosheets of MoS_2_ and ReS_2_, forming a hybrid structure. MRR exhibits excellent absorption performance, with its strongest reflection loss reaching −51.15 dB at 2.0 mm when the filling ratio is only 10 wt%. Meanwhile, the effective absorption bandwidth covers the range of 5.5–18 GHz. Compared to MXene/MoS_2_ composites, MRR with a MoS_2_-ReS_2_ heterogeneous interface exhibits stronger polarization loss ability and superior absorption efficiency at the same thickness. This study provides a reference for the design of transition metal disulfides-based absorbing materials.

## 1. Introduction

Electromagnetic waves play a core role as information carriers in wireless communication technology, bringing convenience to human production activities and lifestyles, and promoting social development and progress. Among them, electromagnetic waves in the microwave band have the characteristics of large information capacity, long transmission distance, wide frequency band, and high efficiency, and are widely used in various communication networks and devices, such as mobile phones, radar detection, near-field communication technology, etc. [1,2,3,4]. Especially, with the rise and promotion of fifth-generation communication technology and new mobile terminals, various microwave communication devices have gradually flooded every corner of production and life. But, at the same time, a large number of microwave communication technology applications can also cause electromagnetic pollution to electronic devices and the surrounding environment and even endanger human health [5,6,7]. In response to the increasing demand for a safe electromagnetic environment, microwave absorbers that can convert incident electromagnetic wave energy into other energy to dissipate have received increasing attention and research [8,9,10].

In recent years, the application of various novel nanomaterials as the main medium to obtain high-performance absorbers through reasonable structural design has gradually become a hot research topic in the field of microwave absorption. Two-dimensional nanomaterials, such as graphene, transition metal disulfides (TMDs), and transition metal carbides/nitrides (MXene), have received widespread attention and research due to their high specific surface area, low density, and good dielectric properties [11,12,13,14,15]. Among them, two-dimensional MXene, represented by Ti_3_C_2_T_x_, possesses metallic dielectric properties and can attenuate electromagnetic wave energy through conductive loss, making it one of the ideal absorbing media [16,17,18]. On the other hand, the high dielectric properties of MXene can also easily lead to impedance mismatch issues. Compounding MXene with other dielectric materials through interface engineering is an effective way to adjust its absorption performance [19,20]. Another two-dimensional medium TMD, with MoS_2_ semiconductor properties as the most typical, has been proven to be able to balance the dielectric properties of MXene [21,22,23]. Unfortunately, there are few reports on the research of absorbers with other TMDs (beyond MoS_2_) and MXene composite structures.

In order to further improve the performance of TMD-based composite absorbers, we have prepared MXene/MoS_2_-ReS_2_ (MMR) composites by loading the hybrid of MoS_2_ and ReS_2_ on the surface of multi-layer MXene, and studied the effect of the TMD hybrid on the performance of absorbers. ReS_2_ is a semiconductor medium with a lattice structure similar to MoS_2_, which has been proven to be able to form heterogeneous interfaces with MoS_2_ [24,25,26]. Therefore, it has been selected as the object of interface engineering for the construction of a MoS_2_-based heterostructure. The successful encapsulation of a MoS_2_-ReS_2_ hybrid on the surface of MXene was demonstrated using electron microscope images and spectroscopic characterization. The electromagnetic characteristics and absorption performance of MoS_2_ and ReS_2_ at different addition ratios are systematically studied. Moreover, the microwave absorption mechanism of MMR and the affection of the MoS_2_-ReS_2_ hybrid are analyzed.

## 2. Experimental Details

### 2.1. Preparation of Ti_3_C_2_T_x_ MXene

Multi-layer Ti_3_C_2_T_x_ solution was prepared through the mild etching method. Specifically, 15 mL of deionized water and 45 mL of hydrochloric acid (HCl) were mixed evenly. In total, 3 g of lithium fluoride (LiF) and 3 g Ti_3_AlC_2_ MAX powders were added into the mixture sequentially and stirred for 24 h. Then, the etched product was washed and centrifuged several times until the pH of the solution was neutral. The stable supernatant suspension was collected and used as multi-layer MXene solution. Commercial Ti_3_AlC_2_ MAX powder with 400 mesh was purchased from Jilin 11 Technology Co., Ltd. (Jilin, China). All reagents were of analytical grade purity and used without further purification.

### 2.2. Preparation of MXene/MoS_2_-ReS_2_

MXene/MoS_2_-ReS_2_ (MMR) was synthesized via a facile hydrothermal method. The molybdenum source and rhenium sources for the preparation of MoS_2_ and ReS_2_ are ammonium molybdate tetrahydrate ((NH_4_)_6_Mo_7_O_24_·4H_2_O) and ammonium perrhenate (NH_4_ReO_4_), respectively. In total, 0.176 g (NH_4_)_6_Mo_7_O_24_·4H_2_O and 0.400 g thiourea (SC(NH_2_)_2_) were dissolved in 30 mL Ti_3_C_2_T_x_ solution (2 mg mL^−1^). Then, the different amounts of NH_4_ReO_4_ were added into the solution to obtain MMR with different ReS_2_ amounts. When the addition ratios of Mo element and Re element were 2:1, 1:1, 1:2, and 1:4, the amounts of NH_4_ReO_4_ were 0.134 g, 0.268 g, 0.536 g, and 1.072 g, respectively. For convenience, the corresponding MMR samples were successively denoted as MMR-1, MMR-2, MMR-3, and MMR-4. After that, the solution was sealed in a Teflon-lined autoclave and kept at 220 °C for 10 h. After naturally cooling to room temperature, the as-obtained precipitation was collected and washed. Finally, the MMR composites were obtained through the vacuum drying of the precipitation. MXene/MoS_2_ composite without the participation of ReS_2_ was also prepared for comparison.

**Figure 1:**
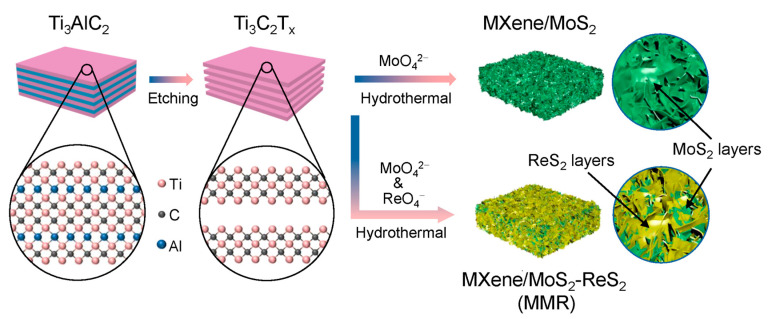
Schematic illustration of the preparation process of MXene/MoS_2_ and MMR samples.

The whole procedure is illustrated in Figure 1.

### 2.3. Evaluation of Properties

The morphologies of samples were observed using scanning electron microscopy (SEM, Inspect F50, FEI). The crystal structures were characterized using X-ray diffraction (XRD, Ultima IV, Rigaku, Tokyo, Japan) with Cu Kα radiation. The molecular vibration was characterized using a Raman spectrum (RAM-PRO-785E, Agiltron, Woburn, MA, USA). The electromagnetic parameters were measured using a Ceyear 3656D vector network analyzer. For the measurement of electromagnetic parameters, the 10 wt% powders of the as-prepared materials were first mixed with 90 wt% paraffin. Here, pure MXene, MXene/MoS_2_, and MMR composites were used for the powder to obtain the corresponding electromagnetic parameters. Then, the mixture was compacted into a coaxial ring with a 7 mm outer diameter and 3.04 mm inner diameter for coaxial testing. The reflection loss (RL) was calculated according to the transmit line theory, as shown below [27]:

(1) $$RL=20\;\log\left|\frac{Z_{in}-Z_{0}}{Z_{in}+Z_{0}}\right|$$

(2) $$Z_{in}=Z_{0}\sqrt{\frac{\mu_{r}}{\varepsilon_{r}}}\tanh\left(j\frac{2\pi fd}{c}\sqrt{\mu_{r}\varepsilon_{r}}\right)$$

where *Z_in_* was the normalized input impedance of the absorber, *Z*_0_ was the impedance of free space, *f* was the frequency of the EM wave, *d* was the thickness of the absorber, and *c* was the velocity of light in free space. *μ_r_* and *ε_r_* were the complex permeability and complex permittivity, respectively.

## 3. Results and Discussion

### 3.1. Structure Analysis

**Figure 2:**
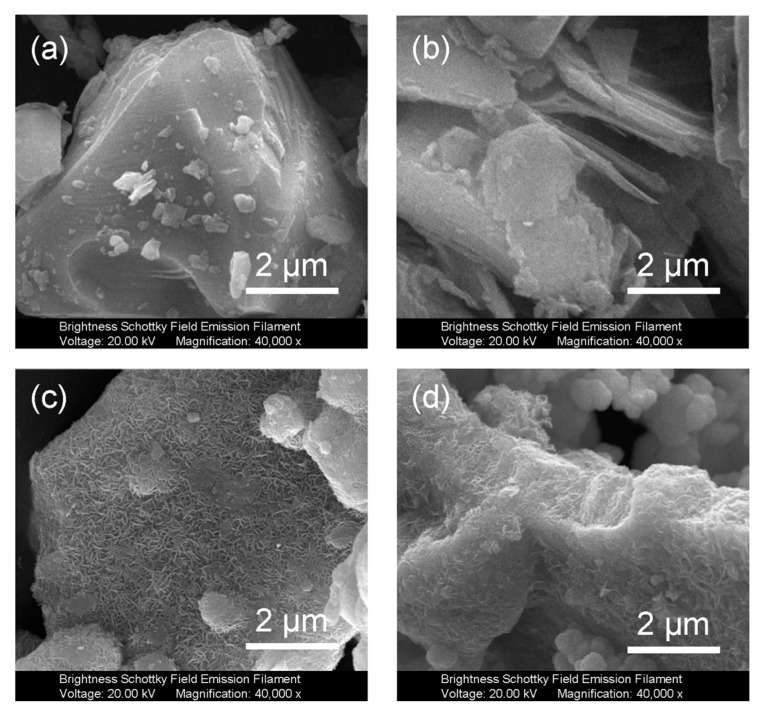
Field-emission scanning electron microscopy (SEM) images of as-obtained samples. (**a**) Ti_3_AlC_2_ MAX, (**b**) Ti_3_C_2_T_x_ MXene, (**c**) MXene/MoS_2_, and (**d**) MMR.

The microstructure morphologies of different samples during the preparation process are shown in Figure 2. The bulk Ti_3_AlC_2_ MAX has a particle size of several micrometers (Figure 2a). After etching, a significant layered phenomenon occurs due to the removal of the Al atomic layer. The multi-layer Ti_3_C_2_T_x_ MXene exhibits a typical accordion-like layered structure, and the particle size of the layers remains at the micrometer level (Figure 2b). After the growth of MoS_2_, the surface of Ti_3_C_2_T_x_ MXene is completely wrapped by MoS_2_ nanosheets, and the outermost layers of MXene/MoS_2_ exhibit an edge-on feature (Figure 2c). The MMR composites containing ReS_2_ exhibit a morphology similar to that of MXene/MoS_2_, with their surfaces completely covered by MoS_2_-ReS_2_ nanosheets (Figure 2d).

**Figure 3:**
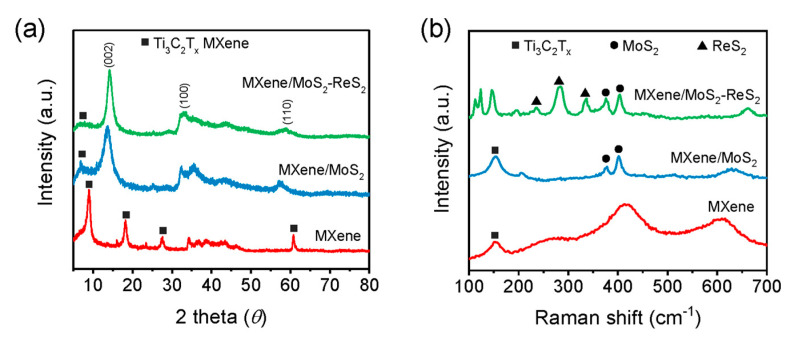
(**a**) XRD patterns and (**b**) Raman spectra of Ti_3_C_2_T_x_ MXene, MXene/MoS_2_, and MMR samples.

To further clarify the structural composition of different samples, XRD was applied to analyze the lattices of Ti_3_C_2_T_x_ MXene, MXene/MoS_2_, and MMR. As shown in Figure 3a, Ti_3_C_2_T_x_ MXene displays four distinct characteristic peaks at 8.9°, 18.2°, 27.6°, and 60.8°, corresponding to (002), (006), (008), and (110) crystal planes, respectively [28]. Among them, the (002) peak shows the highest diffraction intensity, and there is no obvious Ti_3_AlC_2_ MAX characteristic peak, indicating the successful etching of Ti_3_C_2_T_x_ MXene. After growing MoS_2_ nanosheets, MXene/MoS_2_ displays three characteristic peaks at 14.0°, 32.4°, and 57.5°, corresponding to the (002), (100), and (110) crystal planes of 2H-MoS_2_, respectively [29]. The characteristic peak of MXene is significantly weakened in MXene/MoS_2_, indicating that MoS_2_ completely covers the surface and forms a thick shell. It is worth noting that the (002) diffraction peak of Ti_3_C_2_T_x_ MXene shifts towards to 6.9°, as MoS_2_ can grow in the gaps between MXene layers, thereby increasing the spacing of interlayers. Due to the similar sandwich-like lattice structures of ReS_2_ and MoS_2_, the XRD diffraction pattern of MMR is similar to MXene/MoS_2_, exhibiting obvious characteristic peaks near 15°, 33°, and 58°. Therefore, more discriminative characterization is needed to demonstrate the successful loading of ReS_2_ onto MXene.

Considering the different lattice vibration modes of MoS_2_ and ReS_2_, the Raman spectrum can be used for further analysis of MMR, as shown in Figure 3b. Ti_3_C_2_T_x_ MXene exhibits a wide characteristic peak at 152 cm^−1^, which is caused by Ti-O vibration. MXene/MoS_2_ exhibits typical Raman characteristic peaks of MoS_2_ at 378 and 402 cm^−1^, corresponding to in-plane $E_{2g}^{1}$ and the out-of-plane A_1g_ vibration, respectively [30]. These two representative peaks of MoS_2_ can also be preserved in MMR. In addition, new characteristic peaks are observed at 285 cm^−1^ and 336 cm^−1^ in MMR, corresponding to the A_g_-like and E_g_-like vibration modes of ReS_2_ [31], indicating the successful recombination of ReS_2_ onto the MXene surface.

Combining the microscopic morphology images and spectroscopic characterization, it can be proven that the MoS_2_-ReS_2_ hybrid has been successfully grown onto the surface of multi-layer Ti_3_C_2_T_x_ MXene layers. The hybrid structures of MoS_2_ and ReS_2_ exhibit an edge-on nanosheet feature, and uniformly envelop the entire MXene flakes. At the same time, parts of the MoS_2_ and ReS_2_ nanosheets are filled into the gaps between MXene layers, expanding the interlayer spacing of MXene.

### 3.2. Electromagnetic Parameters

**Figure 4:**
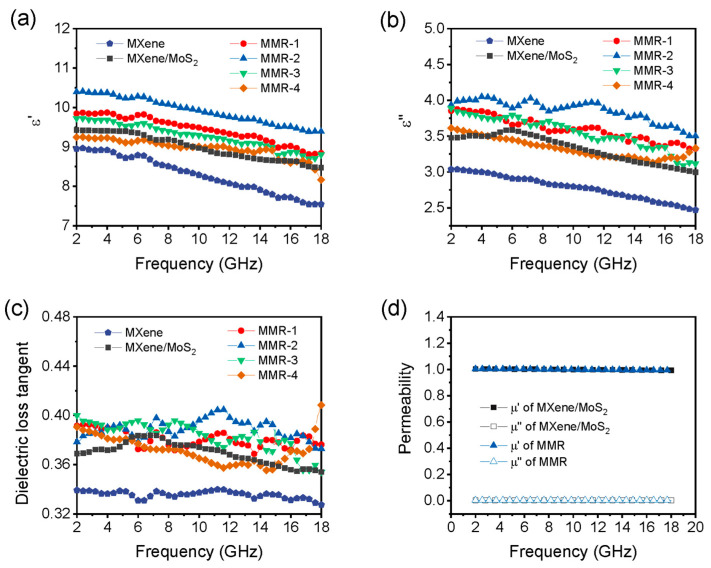
Electromagnetic parameters of pure MXene, MXene/MoS_2_, and MMR samples. (**a**) Real part *ε*′ of complex permittivity, (**b**) imaginary part *ε*″ of complex permittivity, (**c**) dielectric loss tangent of, and (**d**) complex permeability.

The electromagnetic parameters including complex permittivity (*ε*_r_= *ε*′ − *jε*″) and complex permeability (*μ*_r_ = *μ*′ − *jμ*″) of MMR under different ratios of Mo/Re addition, including MMR-1 (2:1), MMR-2 (1:1), MMR-3 (1:2), and MMR-4 (1:4), were measured and compared with MXene/MoS_2_, as shown in Figure 4. All MMR samples and MX/MoS_2_ exhibit higher dielectric constants than pure MXene, indicating that loading TMD on the surface of MXene can simultaneously improve the storage and loss capabilities of electromagnetic waves. The real part of complex permittivity (*ε*′) of MXene/MoS_2_ and all MMR samples deliver a downward trend with increasing frequency in the whole testing range, which is in accordance with Debye theory. The overall value of the *ε*′ first increases to MMR-2 and then decreases. When the ratio of Mo/Re is 1:4, the *ε*′ value of MMR-4 is close to MXene/MoS_2_, and its value in the low-frequency region is actually lower than MXene/MoS_2_. Similarly, the values of the imaginary part of complex permittivity (*ε*″) of MMR exhibit a similar regularity. This indicates that when the ratio of Mo to Re is appropriately increased, the storage and consumption capacity of MMR for electromagnetic wave energy can be improved. When the Mo/Re ratio is 1:1, both the real and imaginary parts of the dielectric constant reach their maximum values. When the proportion of rhenium sources continues to increase from MMR-2 to MMR-3 and MMR-4, the overall electromagnetic wave storage capacity and loss capacity actually decrease. Meanwhile, both the *ε*′′ and the dielectric loss tangent exhibit multiple relaxation peaks, indicating the existence of multiple polarization relaxation processes. Whether it is MXene/MoS_2_ or MMR, its permeability (*μ*_r_ = *μ*′ − *jμ*″) is close to 1 − *j*0, indicating its electromagnetic wave loss mechanism dominated by dielectric loss. This is because no magnetic components are introduced into the entire system, so the real part *μ*′ and imaginary parts *μ*″ of permeability can be taken as 1 and 0, respectively.

### 3.3. Microwave Absorption Performance

**Figure 5:**
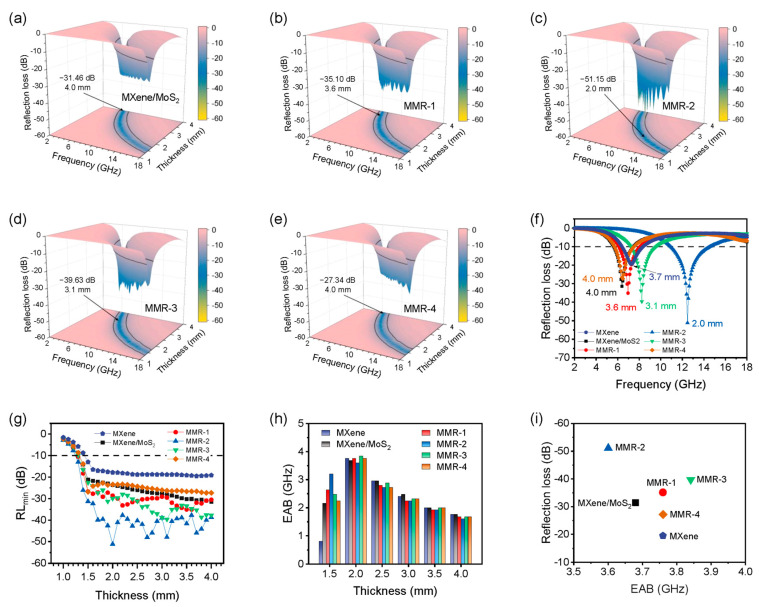
Microwave absorption performance of pure MXene, MXene/MoS_2_, and the MMR series. Three-dimensional RL plots of (**a**) MXene/MoS_2_, (**b**) MMR-1, (**c**) MMR-2, (**d**) MMR-3, and (**e**) MMR-4. (**f**) The optimal RL curves of different samples. (**g**) RL_min_ values of MXene, MXene/MoS_2_, and MMR at different thickness. (**h**) EAB histogram of different samples. (**i**) Comparison of RL_min_ and maximum EAB values of different samples.

Figure 5 shows the microwave absorption performance of MXene/MoS_2_ and the MMR series. From the three-dimensional distribution of RL in Figure 5a–e, it can be seen that the MXene/MoS_2_ and MMR series exhibit similar absorption characteristics on the whole. As the thickness of the absorber increases, the frequency range within which effective absorption (RL < −10 dB) can be achieved, i.e., the effective absorption bandwidth (EAB), shifts towards the low-frequency region. Meanwhile, the EAB region within a thickness range of 1.0 mm to 4.0 mm can cover the frequency range of 6~18 GHz. The strongest reflection loss of MXene/MoS_2_ over the entire frequency range is −31.46 dB, corresponding to a matching thickness of 4.0 mm. When a small amount of ReS_2_ (with the Mo/Re addition ratio of 2:1) is introduced, the absorption performance of the MMR-1 composite system is improved. The strongest reflection loss is increased to −35.1 dB, and the corresponding matching thickness decreases to 3.6 mm. By increasing the addition ratio of Mo/Re to 1:1, MMR-2 can achieve a reflection loss of −51.15 dB at a thickness of 2.0 mm. Continuing to increase the proportion of rhenium will actually result in a decrease in performance to −39.63 dB (MMR-3). The performance of MMR-4 is even inferior to that of MXene/MoS_2_ without ReS_2_ addition.

The optimal RL curves for each sample are shown in Figure 5f. It can be seen that the performance of MMR-2 is far superior to other samples, achieving stronger absorption efficiency at a thickness of 2.0 mm. Figure 5g shows the minimum reflection loss (RL_min_) values of each sample at different thicknesses. The reflection loss of pure MXene under different thicknesses is within −20 dB, and it has been significantly improved after loading with TMD. MMR-2 began to exhibit effective absorption at a thickness of 1.3 mm, while other samples showed 1.4 mm. Under the same thickness, the RL_min_ of MMR-2 is almost entirely greater than that of the other four samples, indicating the excellent absorption capability of MMR-2. Meanwhile, the variation trend of EAB in different samples is also similar, reaching its maximum value at 2.00 mm (Figure 5h). Comparing the RL_min_ and maximum EAB of pure MXene, MXene/MoS_2_, and MMR series (Figure 5i), it can be found that although the widest EAB range of MMR-2 is slightly lower than other samples, the reflection loss and matching thickness possess significant advantages. Therefore, a Mo/Re ratio of 1:1 was selected as the optimal addition ratio. In summary, MMR composites exhibit a significant improvement in microwave absorption efficiency compared to pure MXene and MXene/MoS_2_ while maintaining a close EAB value.

### 3.4. Electromagnetic Wave Response Mechanism

To investigate the influence of the heterogeneous interface in the MoS_2_-ReS_2_ hybrid on the absorption mechanism, a Cole–Cole plot can be used to analyze the polarization behavior [32]. According to Debye’s theory, the dielectric parameters can be described by the following equations:

(3) $$\varepsilon^{\prime }=\varepsilon_{\infty }+\frac{\varepsilon_{s}-\varepsilon_{\infty }}{1+\omega^{2}\tau^{2}}$$

(4) $$\varepsilon^{\prime \prime }=\frac{\varepsilon_{s}-\varepsilon_{\infty }}{1+\omega^{2}\tau^{2}}\omega \tau +\frac{\sigma }{\omega \varepsilon_{0}}$$

where *ω* is the angular frequency, *τ* is the relaxation time, and *σ* is the conductivity. *ε*_s_, *ε*_∞_, and *ε*_0_ are the static dielectric permittivity, relative permittivity at infinite frequency, and dielectric constant in vacuum, respectively. Based on the above equations, without the consideration of *σ*, the relationship between *ε*′ and *ε*′′ can be described by the following equation:

(5) $${\left(\varepsilon^{\prime }-\frac{\varepsilon_{s}+\varepsilon_{\infty }}{2}\right)}^{2}+{\left(\varepsilon^{\prime \prime }\right)}^{2}={\left(\frac{\varepsilon_{s}-\varepsilon_{\infty }}{2}\right)}^{2}$$

**Figure 6:**
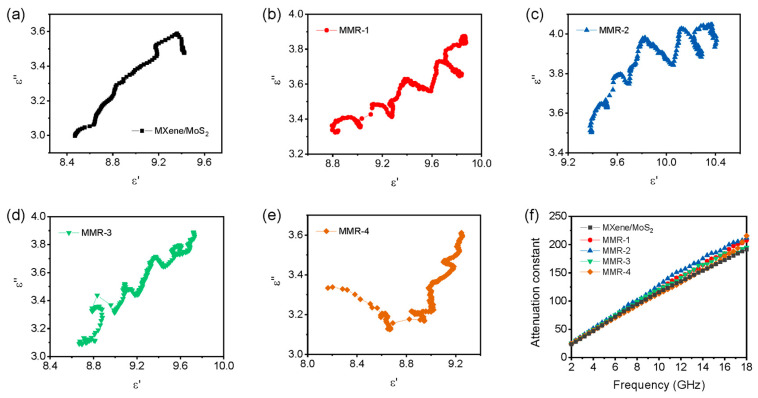
Cole-Cole plots of (**a**) pure MXene and MXene/MoS_2_, (**b**) MMR-1, (**c**) MMR-2, (**d**) MMR-3, and (**e**) MMR-4. (**f**) Attenuation constants of pure MXene, MXene/MoS_2_, and the MMR series.

The curve of *ε*″ versus *ε*′ is depicted as a Cole–Cole curve. A semicircle appears in the Cole–Cole curve and corresponds to one Debye relaxation process. As shown in Figure 6, MXene/MoS_2_ only exhibits significant arc bending in the high-frequency and low-frequency regions. All MMR samples display an undulant curve containing many semicircles, representing the multiple Debye relaxation processes. Compared with MXene/MoS_2_, MMR exhibits significant multiple polarization behavior in the middle-frequency range, which is due to the introduction of ReS_2_, resulting in an additional heterogeneous interface between MXene-ReS_2_ and MoS_2_-ReS_2_. Due to the differences in the dielectric properties of different media, under the action of alternating electric fields, these complex heterogeneous interfaces are conducive to the accumulation of charges at the interface. The changes in the electric field at the interface cannot keep up with the changes in the external electromagnetic field, thus consuming electromagnetic wave energy through polarization relaxation behavior.

Moreover, the attenuation constant (*α*) was applied to evaluate the comprehensive loss ability of MMR microwave absorbers, and its value can be described by the following equation [27]:

(6) $$\alpha =\frac{\sqrt{2}\pi f}{c}\sqrt{\left(\mu^{\prime \prime }\varepsilon^{\prime \prime }-\mu^{\prime }\varepsilon^{\prime }\right)+\sqrt{{\left(\mu^{\prime \prime }\varepsilon^{\prime \prime }-\mu^{\prime }\varepsilon^{\prime }\right)}^{2}+{\left(\mu^{\prime }\varepsilon^{\prime \prime }+\mu^{\prime \prime }\varepsilon^{\prime }\right)}^{2}}}$$

As shown in Figure 6f, the *α* values of pure MXene, MXene/MoS_2_, and MMR exhibit similar trends throughout the whole frequency range, both improving with increasing frequency. In the low-frequency region, the *α* value of MXene/MoS_2_ shows a slight difference in all MMR samples. When the frequency increases to above 10 GHz, the MMR-1 to MMR-3 samples show obvious improvements. MMR-2 exhibits the highest *α* value, indicating its excellent electromagnetic wave-loss ability. The *α* value of MMR-4 is only higher than MXene/MoS_2_ in the range of 15~18 GHz. This can be attributed to the fact that excessive rhenium source addition is not conducive to the growth of MoS_2_-ReS_2_ hybrid on the MXene surface, thereby weakening the overall polarization-loss ability. The results of the *α* value are consistent with the variations in the microwave absorption performance of the MMR series in Figure 5.

**Figure 7:**
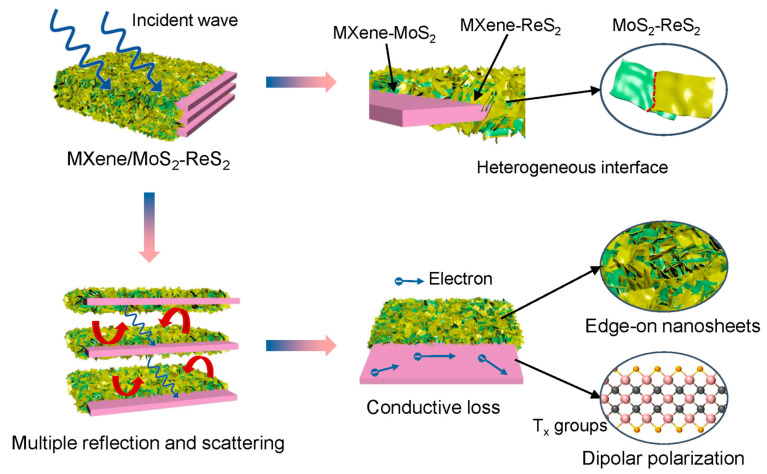
Schematic illustration of microwave absorption mechanisms for the MMR absorber.

Based on the above analysis, the microwave absorption mechanism of MMR can be illustrated in Figure 7. When the incident wave enters the interior of the MMR, due to the presence of complicated heterogeneous interfaces, including MXene and MoS_2_, MXene and ReS_2_, as well as the interfaces between MoS_2_ and ReS_2_, the polarization relaxation process greatly consumes electromagnetic wave energy. Ti_3_C_2_T_x_ MXene owns a two-dimensional layered structure with a high specific surface area, which leads to multiple reflections and scattering in the interior of multi-layer flakes, thereby extending the propagation path of electromagnetic waves and further dissipating electromagnetic wave energy. Meanwhile, owing to the high conductivity of MXene, free electrons can migrate within the layer under alternating electromagnetic fields, converting electromagnetic wave energy into thermal energy through conductive loss. In addition, the abundant functional groups on the terminals of MXene can enhance the dissipation of electromagnetic waves through dipole polarization. It is worth noting that the two-dimensional MoS_2_ and ReS_2_ nanosheets with edge-on morphology are modified on the highly conductive MXene surface, which is beneficial for improving the impedance matching of the absorber and reducing the undesirable reflection of electromagnetic waves on the surface.

## 4. Conclusions

In this paper, the hybrid structures of MoS_2_ and ReS_2_ have been successfully loaded on the surface of Ti_3_C_2_T_x_ MXene layers through interface engineering. By adjusting the addition ratio of molybdenum and rhenium sources (2:1, 1:1, 1:2, and 1:4), MMR absorbers with different absorption characteristics were obtained. Benefiting from the enhanced polarization loss ability by the abundant heterogeneous interface, the absorption performance of MMR is significantly improved compared to MXene/MoS_2_. When the addition ratio of Mo/Re is 1:1, MMR exhibits optimal absorption performance, with the strongest reflection loss increasing from −31.46 dB to −51.15, and the corresponding matching thickness decreasing from 4.0 mm to 2.0 mm. This design strategy of MoS_2_-ReS_2_ heterogeneous interfaces provides a reference for the development of high-performance absorbers based on TMD or MXene.

## Data Availability

The data that support the findings of this study are available on request from the corresponding author.
